# Hahnemannian Association of Pennsylvania

**Published:** 1887-11

**Authors:** 


### HAHNEMANNIAN ASSOCIATION OF PENNSYLVANIA.

A new Society, to be known as "*The Hahnemannian Association of Pennsylvania*," was organized on Tuesday evening, October 11th, at the Continental Hotel, Philadelphia. Like the Lippe Society and kindred organizations, it proposes to deal solely with Homœopathy in its *purity*. Its distinctive feature is a clause of its Constitution requiring that new members shall join the association by degrees—viz.; they are first elected to *associate* membership, in which they have full privilege to enter into debate and receive appointments for work, but cannot hold office nor vote. Before applying for active membership they are required to attend ten stated meetings as associates and to present three original papers. Three negative votes are then necessary to reject their petition to active membership.

The Lippe Society is a more exclusive organization, and is a *social* as well as a medical club. The object of this Association (while friendly to the L. S.) is "strictly business."

The following officers were elected: President, Dr. Mahlon Preston, of Norristown, Pa.; Vice-President, Dr. C. Carleton Smith, Philadelphia; Secretary, Dr. William Jefferson Guernsey, Frankford, Philadelphia; Treasurer, Dr. George H. Clark, Germantown, Philadelphia.

Dr. John V. Allen, of Frankford, Philadelphia, was appointed to prepare an original paper for the next meeting, when Dr. Smith will offer an article on Spongia, and Dr. Clark will give a dissertation on one paragraph of the *Organon.*

Adjourned—to meet in November at the same place.

